# Replacement of Soybean Oil with *Hermetia illucens* Larvae Fat in Layer Diets: Effects on Performance, Egg Quality, Yolk Lipid Composition, and Ovarian Follicles

**DOI:** 10.3390/ani16172661

**Published:** 2026-08-25

**Authors:** Franziska Hornfeck, Ronny Müller, Sarah M. Grundmann, Kristina Maier-Sam, Erika Most, Lea Schäfer, Denise K. Gessner, Klaus Eder

**Affiliations:** 1 Institute of Animal Nutrition and Nutrition Physiology, Justus Liebig University Giessen, Heinrich-Buff-Ring 26-32, 35392 Giessen, Germany; franziskahornfeck@mail.de (F.H.); sarah.grundmann@ernaehrung.uni-giessen.de (S.M.G.); erika.most@ernaehrung.uni-giessen.de (E.M.); denise.gessner@ernaehrung.uni-giessen.de (D.K.G.); 2 Geflügelhof Thierbach GmbH, Thierbach 45, 07368 Remptendorf, Germany; rmueller888@web.de; 3 Clinic for Birds, Reptiles, Amphibians and Fish, Justus Liebig University Giessen, Frankfurter Strasse 114, 35392 Giessen, Germany; kristina.maier@vetmed.uni-giessen.de; 4 Center for Sustainable Food Systems, Justus Liebig University Giessen, Senkenbergstrasse 3, 35390 Giessen, Germany

**Keywords:** *Hermetia illucens*, black soldier fly, laying hens, insect fat, egg quality, fatty acid composition, ovarian follicles, sustainability

## Abstract

This study evaluated the effects of partially (50%) or completely (100%) replacing soybean oil with *Hermetia illucens* larvae fat (HI fat) in diets for laying hens on productive performance, egg quality, yolk lipid composition, and ovarian follicle characteristics during a 20-week feeding period. Sixty Lohmann LSL Classic hens were randomly allocated to three dietary treatments (Control, HI50, and HI100; *n* = 20 hens per treatment). Due to space limitations within the experimental facility, each treatment was housed in a single pen, which precluded statistical analysis of production performance data. Nevertheless, descriptive evaluation revealed no obvious differences in laying rate, egg weight, egg mass, feed intake, or feed conversion ratio among the dietary treatments. Most egg quality traits, including albumen height, Haugh units, shell strength, and yolk color, remained unaffected, although minor transient differences were detected at individual sampling time points. Dietary treatments altered yolk fatty acid composition, reflecting the fatty acid profile of the experimental diets. Replacement of soybean oil with HI fat increased the proportions of saturated fatty acids, particularly lauric (C12:0), myristic (C14:0), and palmitic acids (C16:0), while reducing both *n*-6 and *n*-3 polyunsaturated fatty acids. In conclusion, the findings of this exploratory study indicate that HI fat may have potential as an alternative lipid source in layer diets. Under the conditions of the present experiment, no obvious adverse effects on productive performance, egg quality, or ovarian follicle characteristics were observed. However, because each dietary treatment was represented by a single pen, the results should be interpreted with caution and require confirmation in studies with replicated experimental units.

## Simple Summary

Insect-derived fats are being investigated as more sustainable alternatives to conventional feed ingredients in poultry production. In this study, soybean oil in diets for laying hens was partially or completely replaced with fat from *Hermetia illucens* larvae (HI fat) over a 20-week period. Under the conditions of this exploratory study, no obvious differences were observed in laying performance, feed intake, egg weight, or most egg quality characteristics among the dietary treatments. However, the fatty acid composition of egg yolks changed, reflecting the different fat sources used in the diets. Ovarian follicle characteristics also appeared similar among treatments. Because each dietary treatment was represented by only one replicate group, the results should be interpreted with caution. Nevertheless, the findings indicate that HI fat may have potential as an alternative lipid source for laying hens.

## 1. Introduction

Global demand for animal-derived foods continues to increase as a consequence of population growth and rising living standards, placing considerable pressure on livestock production systems to improve resource efficiency and environmental sustainability [1]. Poultry production plays a pivotal role in meeting the growing demand for animal-derived foods because of its high efficiency in converting feed into high-quality animal protein. [2,3]. Among poultry products, eggs are of particular importance for global food security [4]. Worldwide egg production reached approximately 91 million tonnes in 2023, representing an increase of more than 40% since 2010 [5]. Consequently, layer hen production is expected to contribute increasingly to the supply of affordable and nutritious animal-source foods while simultaneously meeting growing sustainability requirements.

Feed production is a major contributor to the environmental footprint of livestock systems. Concerns about food security and the environmental impacts of conventional feed ingredients have stimulated the search for more sustainable alternative feed resources [6]. Soybean meal and soybean oil remain widely used protein and energy sources in poultry diets; however, soybean cultivation has been associated with deforestation, biodiversity loss, extensive land and water use, and greenhouse gas emissions [7,8]. The development of resource-efficient and locally produced alternatives is therefore receiving increasing attention.

In this context, insect biomass has emerged as a promising feed resource because of its high feed conversion efficiency, ability to utilize low-value organic substrates, and comparatively low environmental footprint [9]. Among the insect species currently considered for livestock feeding, the black soldier fly (*Hermetia illucens*, HI) is of particular interest. Following the authorization of insect proteins for use in poultry and pig diets in the European Union in 2021, HI-derived feed ingredients have become increasingly available for commercial applications [10]. Numerous studies have demonstrated that HI meal can partially replace conventional protein sources in poultry diets without compromising animal performance or product quality [11,12,13,14,15]. These findings are supported by a recent meta-analysis, which concluded that black soldier fly-derived feed ingredients exert overall neutral to positive effects on production performance, meat quality, and physiological characteristics in broilers [16]. 

In addition to protein-rich meal, processing of HI larvae yields a lipid-rich fraction with considerable potential as an alternative feed fat. HI fat differs markedly from conventional vegetable oils due to its relatively high concentration of medium-chain saturated fatty acids, particularly lauric (C12:0) and myristic acid (C14:0) [13,17]. These fatty acids have attracted interest because of their antimicrobial, immunomodulatory, and metabolic properties [18,19,20,21]. Studies in broilers have shown that HI fat can replace conventional lipid sources without adverse effects on growth performance, carcass characteristics, nutrient digestibility, metabolic health or meat quality [22,23,24,25]. 

Interest in the application of HI-derived products in layer nutrition has likewise increased in recent years. Studies by Patterson et al. [26], Kim et al. [27], and Heuel et al. [28] demonstrated that partial or complete replacement of soybean oil with HI-derived fat sources did not markedly affect laying performance or major egg quality traits. In addition, Kumalasari et al. [29] reported beneficial antimicrobial and enzymatic activities following supplementation of layer hen diets with black soldier fly larvae in combination with probiotics. Consistent with these observations, a recent meta-analysis concluded that HI-derived feed ingredients can generally be included in layer diets without substantially affecting production performance or egg quality [30].

Nevertheless, the available evidence in laying hens remains more limited than in broilers. Most studies have focused primarily on productive performance and egg quality and have been conducted over relatively short feeding periods. Consequently, information regarding the long-term effects of high inclusion levels of HI fat, particularly complete replacement of soybean oil, on yolk lipid composition and ovarian characteristics remains limited.

The evaluation of HI fat in laying hens is particularly relevant because dietary lipids play a key role not only in energy supply but also in egg formation and yolk lipid deposition. Alterations in dietary fatty acid composition can directly influence yolk fatty acid profiles and may affect egg quality characteristics such as yolk color and albumen quality [31]. Furthermore, the continuous synthesis of yolk precursors places specific physiological demands on laying hens; therefore, results obtained in growing poultry cannot necessarily be extrapolated to laying birds [32].

Therefore, the objective of the present study was to evaluate the effects of partially (50%) or completely (100%) replacing soybean oil with HI fat in diets for laying hens. The experiment was conducted under practical production conditions over a 20-week feeding period. Particular emphasis was placed on laying performance, egg quality traits, yolk fatty acid composition, and ovarian follicle characteristics. By addressing both productive and physiological responses, the study contributes to the assessment of HI fat as a sustainable alternative lipid source for layer nutrition and supports the development of more resource-efficient feeding strategies for egg production.

## 2. Materials and Methods

### 2.1. Animals and Experimental Design

A total of 60 LSL Classic pullets (18 weeks of age; Lohmann Deutschland, Ankum, Germany) were obtained from a commercial breeder (Brinkschulte GmbH & Co. KG Geflügelzucht, Senden, Germany) and used in this study. All hens were individually identified using colored plastic leg rings and assigned to one of three dietary treatments (*n* = 20 hens per treatment). To avoid selection bias, birds were sequentially selected from the flock and allocated to the treatment groups without regard to body weight or other animal characteristics. As a result, initial body weights were comparable among treatments, with no significant differences at the start of the experiment.

The study was conducted under practical on-farm conditions and was designed as an exploratory feeding trial. Due to space limitations within the experimental facility, only one pen could be provided for each dietary treatment, and replicate pens were therefore not available. As a result, both pen-level and egg-level observations were derived from a single experimental unit per treatment, and treatment effects should be interpreted with caution. The implications of this design for the statistical evaluation of performance traits are described in the Statistical Analysis section.

The experimental diets differed in the source of supplemental dietary fat. The control diet contained soybean oil as the sole added lipid source. In the HI-50 diet, 50% of the soybean oil was replaced by HI fat, whereas in the HI-100 diet soybean oil was completely replaced by HI fat. Apart from the fat source, diets were formulated to be comparable in nutrient composition.

To minimize potential bias, personnel responsible for animal management and feed allocation were aware of the dietary treatments, whereas all individuals involved in data collection, laboratory analyses, and statistical evaluation remained blinded to treatment assignment throughout the study.

### 2.2. Housing and Management

Each treatment group (*n* = 20 hens) was housed in a separate compartment within a floor-housing system. All compartments were identical in design and provided 5.0 m^2^ of floor space. Each compartment was equipped with one circular drinker (diameter: 35 cm), one circular feeder (diameter: 50 cm), four perches with a combined length of 4.5 m, and eight individual nest boxes (28 × 38 × 38 cm; length × width × height). Group size and compartment dimensions were selected to comply with applicable housing and animal welfare regulations and to provide sufficient space for normal behavioral expression.

In addition to natural daylight, flicker-free LED lamps served as the artificial light source to ensure uniform illumination throughout the facility. The lamps were installed at a height of 2.88 m above floor level and provided approximately 20 lux at bird level. The lighting schedule was gradually adjusted during the experimental period in accordance with the management recommendations for LOHMANN LSL-Classic laying hens [33], increasing from 8.5 h of artificial light per day at the beginning of the trial to 16 h per day during the main laying period.

Environmental conditions were maintained using an electrically operated ventilation system, which was manually adjusted according to indoor temperature. During the experimental period (23–40 weeks of age), mean temperature and relative humidity were 20.8 ± 3.7 °C and 61.6 ± 7.7%, respectively. Both parameters were recorded twice daily. Wood shavings were used as litter material to facilitate natural behaviors such as scratching and dust bathing. In addition, pecking stones were provided as environmental enrichment. Fresh drinking water was available at all times; drinkers were cleaned daily and refilled as needed. Housing facilities were cleaned when necessary, and manure was removed from the compartments at intervals of seven to ten days.

### 2.3. Diets

Until the middle of week 19 of age, all hens received a commercial pullet diet, followed by a pre-lay diet for 8 days. Avesgran poultry grit (2–4 mm; Euroquarz GmbH, Laußnitz, Germany) was provided ad libitum throughout the entire experimental period. The experimental diets were fed in two phases: phase 1 from week 20 to week 30 of age and phase 2 from week 31 to week 40 of age.

The three dietary treatments were formulated to meet the nutrient requirements of laying hens and differed primarily in the source of supplemental fat. The control diet contained soybean oil as the sole added lipid source (65 g/kg diet). In the HI-50 diet, 50% of the soybean oil was replaced by HI fat, whereas soybean oil was completely replaced by HI fat in the HI-100 diet. Because the HI fat contained less fat and metabolizable energy than soybean oil, slightly higher inclusion levels of HI fat were required to provide comparable amounts of dietary lipid. Consequently, minor adjustments were made to the inclusion levels of other ingredients, including wheat, corn, and wheat bran, to maintain comparable dietary energy and nutrient concentrations across treatments. The ingredient composition of the experimental diets is shown in Table 1.

All diets were prepared individually under standardized conditions. To ensure a homogeneous distribution of ingredients, components with low inclusion rates were first incorporated into a premix before being combined with the remaining ingredients. Subsequently, the complete mixtures were blended for 45 min in a 100 kg mixer (Dierks & Söhne GmbH & Co. KG, Osnabrück, Germany). The mixer was equipped with a lid to minimize feed losses and prevent ingredient segregation during processing.

The HI fat used in this study was supplied by GmbH (Pegau, Germany). According to the manufacturer, the fat was obtained by mechanical extraction. Briefly, HI biomass was dried to a dry matter content of 90–95%, heated to approximately 80 °C, and subsequently pressed using a screw press (Reinartz GmbH & Co. KG, Neuss, Germany) to separate the fat fraction.

The HI fat contained 89.5% ether extract and was characterized by a high proportion of lauric acid (C12:0; 57.2 g/100 g total fatty acids), followed by palmitic acid (C16:0; 10.9 g/100 g total fatty acids), linoleic acid (C18:2 n-6; 10.6 g/100 g total fatty acids), and myristic acid (C14:0; 8.7 g/100 g total fatty acids). Soybean oil was obtained from Curo Spezialfutter GmbH & Co. KG (Ennigerloh, Germany). Replacement of soybean oil with HI fat was performed on an ether extract basis. All experimental diets were formulated to be isoenergetic and isonitrogenous and contained comparable total fat concentrations (Table 1). Metabolizable energy content was calculated according to the WPSA prediction equation described in Section 2.4. 

Both lipid sources were stored at −20 °C until use. Diets were prepared shortly before the respective feeding phases and subsequently stored at 7 °C until feeding. Feed and water were offered ad libitum throughout the study.

### 2.4. Analysis of Feed Composition

Dry matter, crude ash, crude protein, ether extract, crude fiber, sugar, and starch concentrations in feed ingredients and experimental diets were determined according to the official methods of the Verband Deutscher Landwirtschaftlicher Untersuchungs und Forschungsanstalten (VDLUFA) [34].

Amino acid concentrations in feed ingredients and diets were analyzed by ion-exchange chromatography following post-column derivatization with ninhydrin [35,36]. Briefly, samples were oxidized with performic acid, which was subsequently neutralized with sodium metabisulfite. Amino acids were then released from protein by hydrolysis with 6 N hydrochloric acid for 24 h at 110 °C and quantified using an internal standard. Detection was based on spectrophotometric measurement of the ninhydrin reaction products at 570 nm.

Crude protein content was calculated from total nitrogen using a conversion factor of 6.25. Apparent metabolizable energy corrected for nitrogen (AMEn) was estimated according to the equation recommended by the World’s Poultry Science Association [37]:

AME_N_ (MJ/kg) = (0.01551 × crude protein) + (0.03431 × ether extract) + (0.01669 × starch) + (0.01301 × sugar)

### 2.5. Recording of Feed Intake, Body Weight, Egg Production, and Egg Mass

Prior to the start of the experiment, all hens were individually marked with leg bands. For animal welfare reasons, hens were not fasted prior to weighing, and body weight measurements were limited to the beginning and the end of the trial to minimize handling stress. Individual body weights were measured at these two time points using a digital poultry scale (BAT1, VEIT Electronics, Moravany, Czech Republic). Feed intake was recorded daily at the pen level by weighing feed residues from the previous day.

Eggs were collected and counted daily for each treatment group from the onset of lay (19–20 weeks of age). From week 23 onward, laying rate (%) was calculated as the daily number of eggs produced relative to the number of hens in each group. Egg weight was determined daily using an electronic balance (KERN EHA 3000-1, KERN & Sohn GmbH, Balingen, Germany), and daily egg mass was calculated accordingly.

### 2.6. Assessment of Egg Quality Traits

In weeks 25, 30, 35, and 40 of age, eggs from a single daily collection were sampled from each treatment group and analyzed for internal and external quality traits on the following day. The number of eggs examined per sampling occasion ranged from 18 to 20, depending on the number of hens that laid an egg on the day of collection. Until analysis, eggs were stored at room temperature (18.5–20.0 °C).

The evaluated egg quality traits included shell breaking strength, yolk color, albumen height, and Haugh units. Shell breaking strength was measured by static compression along the longitudinal axis (pole-to-pole orientation) using a shell strength tester (BMG mc 1.2, Messtechnik Gutsch, Nauenhof, Germany), and the force required to fracture the shell was recorded in Newtons (N).

Yolk color was assessed after breaking the eggs onto white porcelain plates and scored visually using the Hoffmann–La Roche color fan (scale 1–15) under standardized lighting conditions. All evaluations were conducted by a single trained assessor who was blinded to treatment allocation, with samples identified only by numerical codes.

Albumen height was measured with an analog albumen height gauge (Baxlo Haugh Micrometer, Baxlo Mess- und Präzisionsinstrumente, Polinyà, Spain). Measurements were taken at three locations approximately 1 cm from the yolk margin, and the mean value was used for further evaluation. Haugh units (HU), an indicator of albumen quality, were subsequently calculated from albumen height and egg weight according to the standard formula.

### 2.7. Lipid Analysis of Egg Yolk

For yolk lipid analyses, 10 eggs per treatment were randomly selected from the eggs collected within each treatment group at weeks 30 and 40. Yolk triglyceride and cholesterol concentrations were determined enzymatically as described by Eder and Kirchgessner [38]. Briefly, 25 mg of homogenized yolk sample was extracted with 10 mL hexane/isopropanol (3:2, *v*/*v*) for 18 h. For triglyceride and cholesterol analyses, aliquots of 25 µL and 300 µL, respectively, were evaporated to dryness at 45 °C. The extracted lipids were subsequently dissolved in Triton X-100/chloroform (1:1, *v*/*v*), re-evaporated, and quantified photometrically at 500 nm and 37 °C using commercial enzymatic assay kits (DiaSys Diagnostic Systems, Holzheim, Germany; triglyceride kit no. 157109910023; cholesterol kit no. 113009910023). Calibration was performed using triglyceride and cholesterol standards, and assay performance was verified daily using Contronorm quality-control material.

The fatty acid composition of dietary fats and total yolk lipids was determined by gas chromatography. Briefly, 100 mg of dietary fat or 150 mg of homogenized yolk sample was extracted with hexane/isopropanol (3:2, *v*/*v*), using nonadecanoic acid (C19:0) as an internal standard. Following transesterification to fatty acid methyl esters (FAME) by boron trifluoride-methanol, samples were analyzed using a PerkinElmer Clarus 580 gas chromatograph (PerkinElmer, Waltham, MA, USA) equipped with a polar capillary column (30 m × 0.25 mm × 0.25 µm) and a flame ionization detector. Helium served as the carrier gas. The chromatographic conditions and temperature program are described in detail by Schlegel et al. [39]. Individual FAMEs were identified by comparing retention times with those of a commercial FAME standard mixture and were quantified relative to the internal standard.

### 2.8. Recording and Measurement of Ovarian Follicles

The abdominal cavity was opened by a midline incision, and the left ovary was exposed, carefully dissected from surrounding tissues, and removed. The left ovary was selected for assessment because it represents the functional ovary in adult hens, while the right ovary undergoes regression during embryonic development and remains vestigial in mature birds. All visible ovarian follicles exceeding 10 mm in diameter were counted.

The longitudinal and transverse diameters of each follicle were measured to the nearest millimeter using a graduated ruler. Given the relatively large size of the follicles (>10 mm) examined and the exploratory nature of the study, measurement precision to the nearest millimeter was considered adequate. All ovarian assessments were conducted by the same observer.

### 2.9. Animal Health and Welfare

Animal health and welfare were monitored throughout the experimental period using a standardized assessment protocol. In addition to inspections performed at least twice daily, weekly welfare evaluations assessed general appearance, behavior, feed and water intake, and productive performance. A camera-based surveillance system allowed continuous monitoring outside routine husbandry periods. Veterinary supervision was provided throughout the study by a licensed veterinarian (F.H.). In addition to routine daily inspections, a comprehensive health and welfare assessment based on a standardized scoring sheet was conducted weekly. Predefined exclusion criteria included the occurrence of clinical signs of disease or other visible abnormalities, whereas marked reductions in feed intake or egg production were defined as study termination criteria. No animals met the exclusion criteria, and none of the termination criteria were reached during the study. No mortality, clinical signs of disease, or other health-related abnormalities were observed during the experimental period.

In week 11 of the experiment, all hens were vaccinated against Newcastle disease using an attenuated live vaccine (AviPro^®^ ND LASOTA, ELANCO GmbH, Cuxhaven, Germany) administered via the drinking water. No additional vaccinations were performed during the study. To maintain biosecurity, access to the experimental facility was restricted to personnel directly involved in animal care and veterinary supervision and was only possible through a hygiene barrier system operating according to the clean-dirty (“black-white”) principle. Dedicated protective clothing was worn, and footwear and hands were disinfected before entering the facility. The housing unit was equipped with a separate ventilation system, and routine cleaning procedures, rodent monitoring, and preventive pest control measures were implemented throughout the study. Feed was stored in a rodent-proof refrigerated storage room and litter material in a separate enclosed building. Drinking water was supplied from the public potable water network.

### 2.10. Statistical Analysis

Statistical analyses were performed using SPSS version 29 (IBM Corp., Armonk, NY, USA) and R version 4.5.1 (R Foundation for Statistical Computing, Vienna, Austria). Because all hens within a treatment group were housed in a single compartment, the pen represented the experimental unit for performance-related variables, including laying rate, feed intake, feed conversion ratio, egg weight, and egg mass. Consequently, only one experimental unit was available per treatment, precluding inferential statistical comparisons in the classical sense. 

To descriptively explore potential treatment-related differences in egg production, egg weight and daily egg mass, generalized least squares (GLS) models with a first-order autoregressive [AR(1)] correlation structure were fitted to the data from 23 to 40 weeks of age using the R package nlme. Treatment, week of age, and their interaction were included as fixed effects, with the treatment × week interaction used to assess differences in temporal patterns among treatments. Model diagnostics included assessment of residual normality, homogeneity of variance, and residual autocorrelation. Egg mass showed a borderline violation of normality (Shapiro–Wilk *p* = 0.046), attributable to a single influential observation in the control group, which did not substantially affect the overall pattern. The absence of pen replication limits the interpretation of these analyses, and results for production parameters should therefore be considered exploratory.

For body weight, ovarian follicle measurements, egg quality traits, and yolk lipid composition, individual hens or eggs served as the sampling units (*n* = 12–20 per treatment). Although individual observations originated from a single pen per treatment and therefore cannot be considered fully independent experimental units, analyses at the hen or egg level were considered informative because the variables were measured on individual animals or eggs and reflected biological responses at the individual level. Nevertheless, because treatment and pen effects were confounded, the resulting statistical inferences should be regarded as exploratory and interpreted with appropriate caution.

Data were assessed for normality using the Shapiro–Wilk test *(n* < 50) or the Kolmogorov–Smirnov test (*n* ≥ 50). Homogeneity of variances was evaluated using Levene’s test. When normality was achieved only after logarithmic transformation, the transformed data were used for statistical analyses.

Normally distributed data with homogeneous variances were analyzed by one-way analysis of variance (ANOVA), followed by Fisher’s least significant difference (LSD) test for pairwise comparisons when the overall *F*-test was significant. In cases of heterogeneous variances, Welch’s ANOVA and the Games–Howell post hoc test were applied. Non-normally distributed data were analyzed using the Kruskal–Wallis test, followed by pairwise Mann–Whitney U tests with Bonferroni adjustment.

Statistical significance was declared at *p* < 0.05.

## 3. Results

### 3.1. Performance Data 

Throughout the experimental period, no clinical signs of disease, external abnormalities, or mortality were observed in any treatment group. Hens exhibited normal behavior throughout the study, and feed and water consumption remained within the expected physiological range under all dietary treatments.

Initial and final body weights, as well as body weight gains, are presented in Table 2. Production-related parameters, including laying rate, egg weight, daily egg mass, feed intake, and feed conversion ratio, are summarized in Table 3. Owing to the experimental design, production parameters could not be subjected to formal statistical analysis and are therefore presented descriptively.

Initial body weights did not differ among treatment groups (Table 2). Likewise, final body weights and body weight gains were not affected by dietary treatment, indicating that partial or complete replacement of soybean oil with HI fat had no detectable effect on body weight development during the experimental period.

Egg production rate, feed intake, and feed conversion ratio were numerically higher in the HI-50 and HI-100 groups than in the control group (Table 3). 

**Figure 1:**
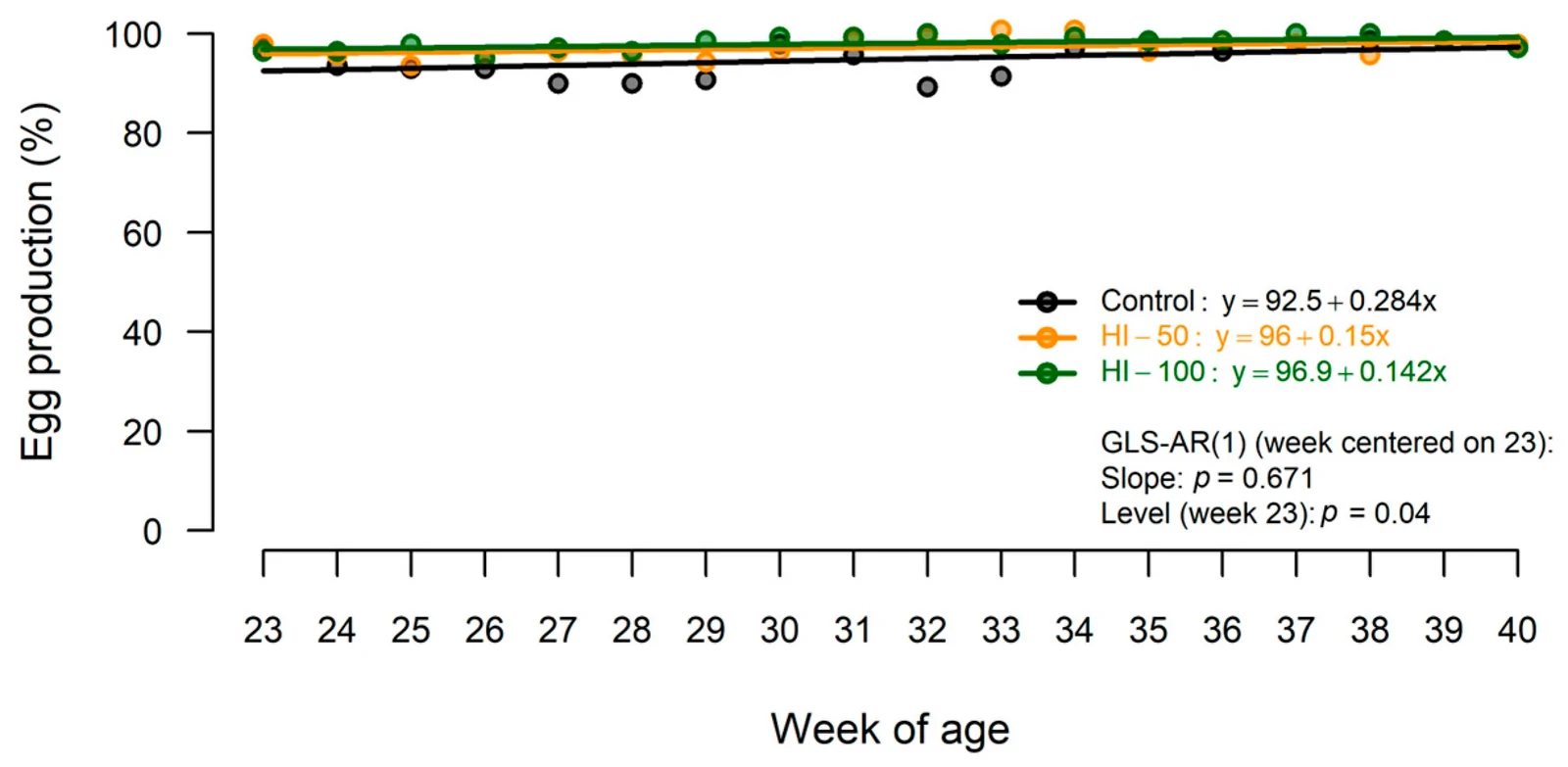
Temporal development of egg production (%) in laying hens fed either a conventional diet containing soybean oil as the primary fat source (Control, C) or diets in which 50% (HI-50) or 100% (HI-100) of soybean oil was replaced with *Hermetia illucens* (HI) larvae fat from 23 to 40 weeks of age. Points represent weekly mean egg production values for each treatment group, and solid lines indicate fitted values from generalized least squares (GLS) models with an AR(1) correlation structure. Differences in temporal patterns among groups were evaluated using GLS-AR(1), with week as a continuous covariate. The slope *p*-value represents the group × week interaction, whereas the level *p*-value represents differences in intercepts. All hens within a treatment were housed in a single compartment.

To explore whether dietary treatment influenced the development of laying performance over time, egg production rate was additionally evaluated using GLS AR(1). At the beginning of this period (week 23), egg production was higher in the HI-50 and HI-100 groups than in the control group (*p* < 0.05), as reflected by differences in the regression intercepts. In contrast, the slopes of the regression lines did not differ among treatments (Figure 1), indicating that egg production developed similarly across all treatment groups throughout the experimental period. Collectively, these observations provide no indication that partial or complete replacement of soybean oil with HI fat adversely affected laying performance.

**Figure 2:**
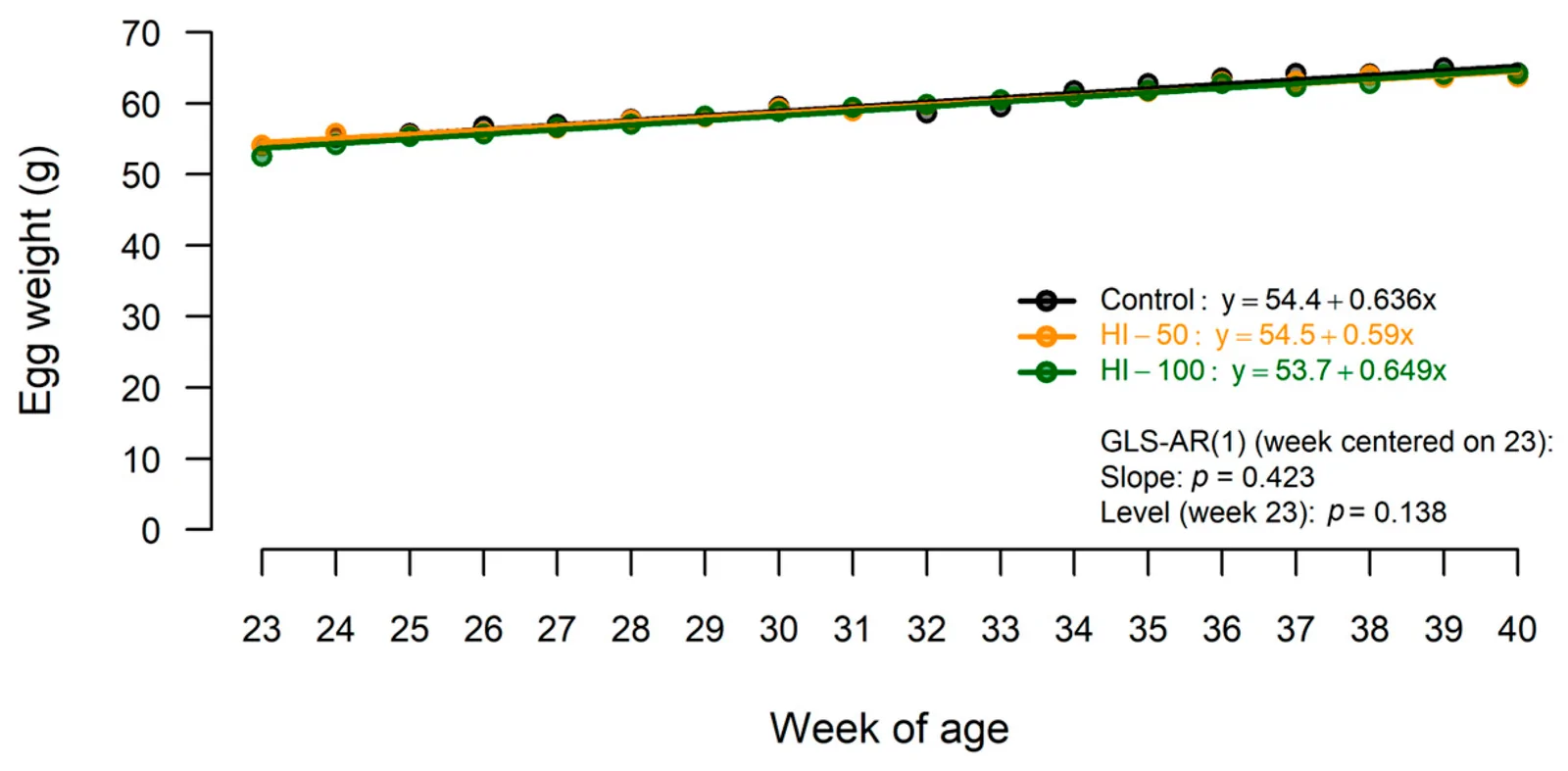
Temporal development of egg weight (g) in laying hens fed either a conventional diet containing soybean oil as the primary fat source (Control, C) or diets in which 50% (HI-50) or 100% (HI-100) of soybean oil was replaced with *Hermetia illucens* (HI) larvae fat from 23 to 40 weeks of age. Points represent weekly mean egg weights for each treatment group, and solid lines indicate fitted values from generalized least squares (GLS) models with an AR(1) correlation structure. Differences in temporal patterns among groups were evaluated using GLS-AR(1), with week as a continuous covariate. The slope *p*-value represents the group × week interaction, whereas the level *p*-value represents differences in intercepts. All hens within a treatment were housed in a single compartment.

**Figure 3:**
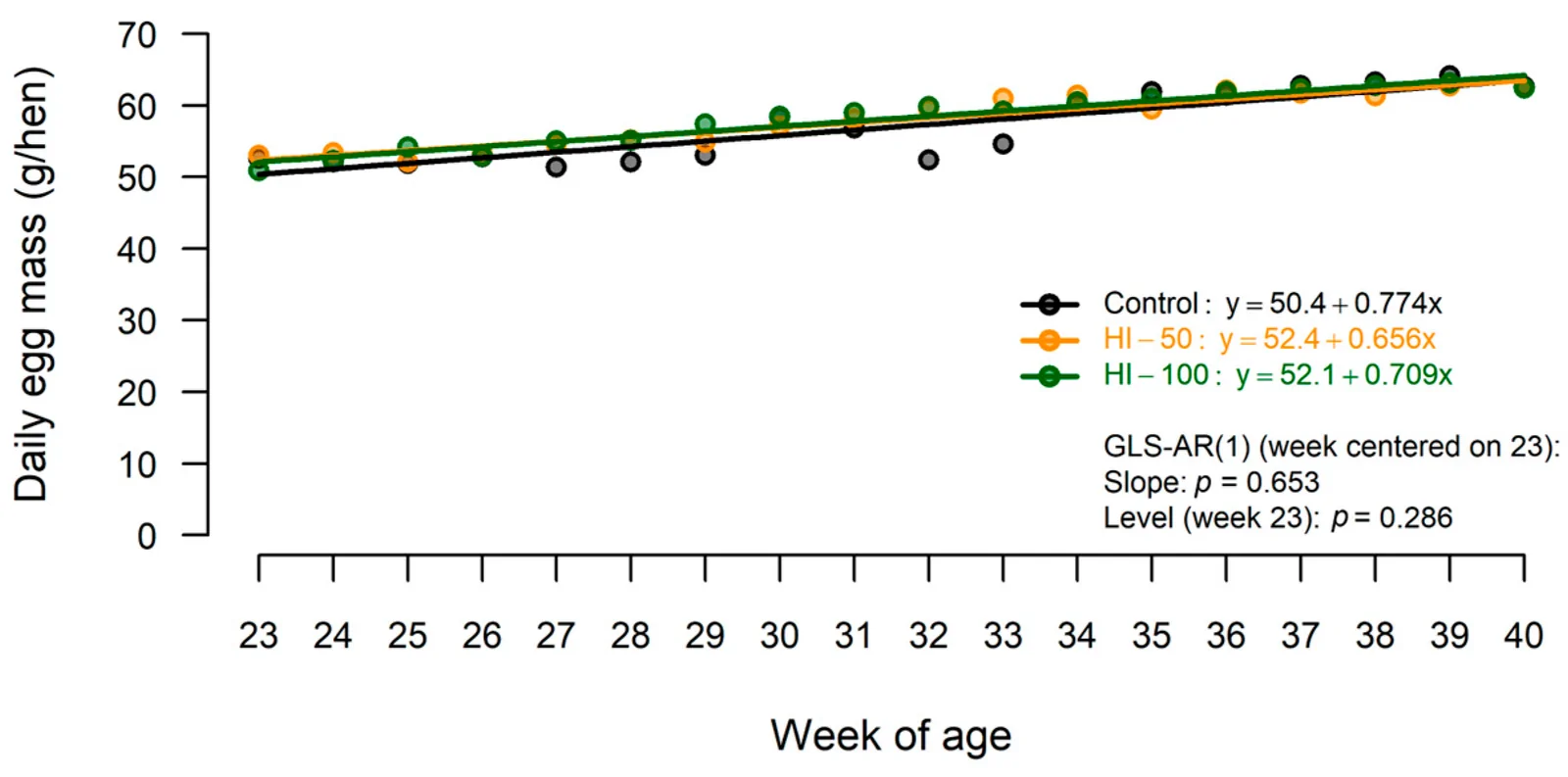
Temporal development of daily egg mass (g/hen) in laying hens fed either a conventional diet containing soybean oil as the primary fat source (Control, C) or diets in which 50% (HI-50) or 100% (HI-100) of soybean oil was replaced with *Hermetia illucens* (HI) larvae fat from 23 to 40 weeks of age. Points represent weekly mean daily egg mass for each treatment group, and solid lines indicate fitted values from generalized least squares (GLS) models with an AR(1) correlation structure. Differences in temporal patterns among groups were evaluated using GLS-AR(1), with week as a continuous covariate. The slope *p*-value represents the group × week interaction, whereas the level *p*-value represents differences in intercepts. All hens within a treatment were housed in a single compartment.

Mean egg weight was numerically comparable among the three groups, while daily egg mass was numerically greater in the HI-50 and HI-100 groups than in the control group (Table 3). GLS analyses with an AR(1) correlation structure over the period from 23 to 40 weeks of age showed similar temporal patterns for egg weight (Figure 2) and daily egg mass (Figure 3) across treatments. Thus, replacing soybean oil with HI fat did not appear to adversely affect egg weight or egg mass production over the experimental period.

### 3.2. Egg Quality Traits 

Egg quality traits are summarized in Table 4. Albumen height and Haugh units differed among treatments at 25 weeks of age, with hens in the HI-50 group exhibiting higher values than those in the HI-100 group, while the control group showed intermediate values. However, no significant treatment effects on albumen height or Haugh units were detected at 30, 35, or 40 weeks of age.

Shell breaking strength was not affected by dietary treatment at any sampling point. In contrast, yolk color scores differed among treatments at 30 and 35 weeks of age, with the HI-100 group showing higher values than the control group and the HI-50 group displaying intermediate values. No significant differences in yolk color were observed at 25 or 40 weeks of age.

Overall, the effects of dietary treatment on egg quality traits were limited and not consistently observed across the experimental period. Apart from the temporary differences in albumen quality and yolk color at individual sampling points, replacement of soybean oil with HI fat did not markedly affect egg quality parameters. In addition, statistical comparisons were performed separately at each sampling time point, and no adjustment for multiple testing across the four repeated measurements per trait was applied. Therefore, the isolated significant differences observed at individual time points should be interpreted with caution.

### 3.3. Fatty Acid Composition of Yolk Total Lipids and Lipid Contents in Egg Yolk 

The fatty acid composition of yolk total lipids at 30 and 40 weeks of age is presented in Table 5. At both sampling times, replacing soybean oil with HI fat markedly altered the fatty acid profile of egg yolks. Specifically, the proportions of lauric acid (C12:0), myristic acid (C14:0), and palmitic acid (C16:0) increased, resulting in a higher proportion of total saturated fatty acids (SFA) in the HI-fed groups. In contrast, the proportion of stearic acid (C18:0) decreased.

Among the monounsaturated fatty acids (MUFA), the proportion of palmitoleic acid (C16:1) increased with HI fat inclusion, whereas oleic acid (C18:1 n-9) remained unaffected. Consequently, the proportion of total MUFA was higher in the HI-fed groups than in the control group.

Replacement of soybean oil with HI fat also led to reductions in the proportions of linoleic acid (C18:2 n-6), α-linolenic acid (C18:3 n-3), and docosahexaenoic acid (C22:6 n-3), resulting in lower concentrations of total n-6 and n-3 polyunsaturated fatty acids (PUFA). Despite these changes in individual fatty acids and PUFA concentrations, the ratio of total n-6 to n-3 PUFA remained unaffected by dietary treatment at both 30 and 40 weeks of age.

Concentrations of triglycerides and cholesterol in egg yolks are shown in Table 6. At 30 weeks of age, yolk triglyceride concentrations were lower in the HI-100 group than in the control group (*p* < 0.05), while the HI-50 group exhibited intermediate values. In the same sampling period, yolk cholesterol concentrations were lower in the HI-50 group than in both the control and HI-100 groups (*p* < 0.05). No significant differences in either triglyceride or cholesterol concentrations were detected among treatments at 40 weeks of age. Overall, treatment effects on yolk triglyceride and cholesterol concentrations were not persistent across sampling times.

### 3.4. Number and Size of Ovarian Follicles

**Figure 4:**
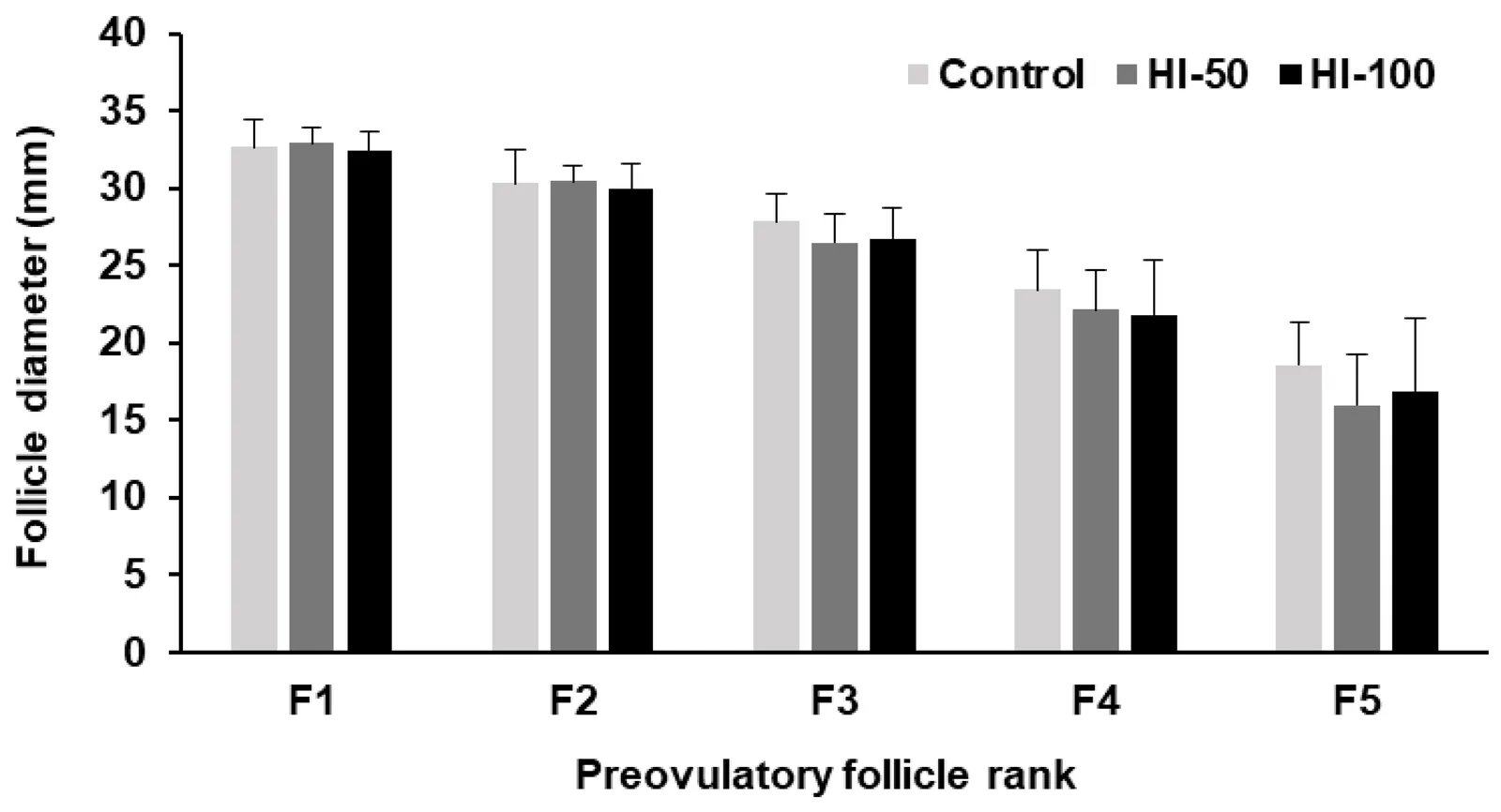
Follicle diameters (mm) of preovulatory follicles (F1–F5) in laying hens fed either a conventional diet containing soybean oil as the primary fat source (Control) or diets in which 50% (HI-50) or 100% (HI-100) of soybean oil was replaced with *Hermetia illucens* (HI) larvae fat. Bars represent mean follicle diameters, and error bars indicate standard deviations. Data were obtained from follicles collected from *n* = 12 hens per treatment group. Follicles were classified according to hierarchical preovulatory follicle rank (F1 = largest, most mature follicle; F5 = fifth largest follicle). All hens within each treatment were housed in a single compartment.

**Figure 5:**
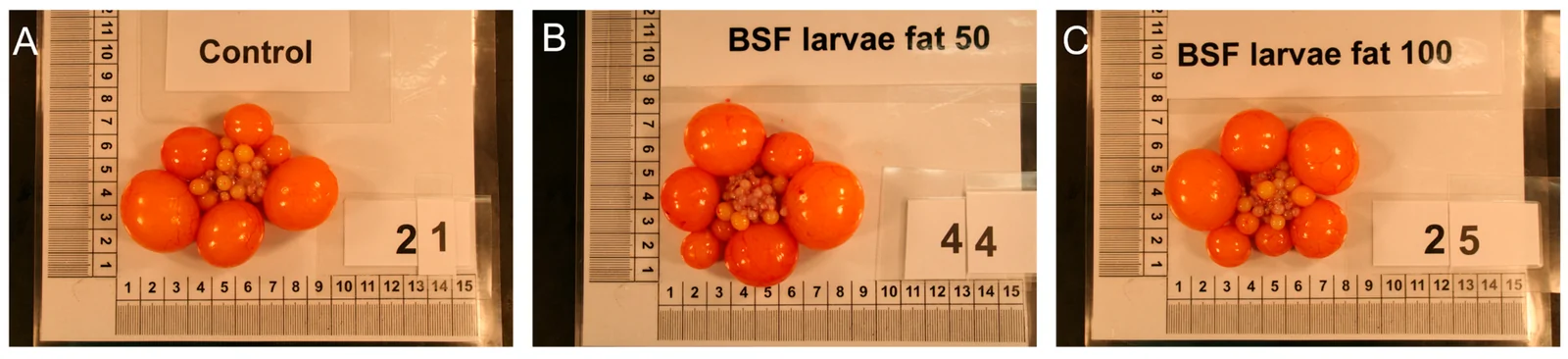
Representative images of ovarian follicles from laying hens in the control group (**A**), HI-50 group (**B**), and HI-100 group (**C**), fed either a conventional diet containing soybean oil as the primary fat source or diets in which 50% or 100% of soybean oil was replaced with *Hermetia illucens* (HI) larvae fat. The images illustrate typical macroscopic appearance of ovarian follicles at the time of sampling. All hens within each treatment were housed in a single compartment.

The number of ovarian follicles with a diameter ≥10 mm was not affected by dietary treatment (control: 5.92 ± 0.90; HI-50: 5.83 ± 0.72; HI-100: 6.42 ± 1.08; means ± SD; *p* > 0.05). Similarly, follicular diameters did not differ among groups (Figure 4). Thus, neither the number nor the size of ovarian follicles was influenced by partial or complete replacement of soybean oil with *Hermetia illucens* fat. Representative ovarian images from one hen per treatment are presented in Figure 5. 

## 4. Discussion

The aim of the present study was to evaluate whether soybean oil in layer hen diets can be partially or completely replaced by fat derived from HI larvae without adversely affecting productive performance, egg quality, or indicators of animal health, while inducing the expected modifications in yolk fatty acid composition.

A major limitation of this study is that each dietary treatment was represented by a single pen. Consequently, treatment effects were completely confounded with potential pen effects, preventing a formal separation of dietary and pen-related sources of variation. The results should therefore be regarded as exploratory rather than confirmatory. This limitation applies not only to production traits but also to egg quality measurements, yolk lipid composition, and ovarian follicle characteristics, as all observations originated from hens housed within a single pen per treatment. Individual hens and eggs should therefore be considered subsamples of a single experimental unit rather than independent biological replicates. Although statistical analyses were performed at the hen or egg level, the lack of pen replication limits statistical inference, and *p*-values reported for hen- or egg-level measurements should not be interpreted as definitive evidence of treatment effects. Accordingly, the present findings should be interpreted as indicative of biological responses and treatment-related trends that require confirmation in studies employing replicated experimental units.

### 4.1. Laying Performance

The similar temporal pattern of egg production observed among treatments indicates that dietary inclusion of HI fat did not adversely affect laying performance over the 20-week experimental period. The observations obtained in the present study are consistent with a recent meta-analysis indicating that black soldier fly-derived feed ingredients generally do not adversely affect laying performance or egg quality in laying hens [30]. Consistent with the observations obtained in the present study, Patterson et al. [26], Kim et al. [27], and Heuel et al. [28] reported that soybean oil can be partially replaced by HI-derived lipid sources without impairing laying performance. Patterson et al. [25] replaced 4.17% soybean oil with HI fat during a 4-week feeding period, whereas Kim et al. [26] and Heuel et al. [27] evaluated HI fat or HI meal-derived fat over feeding periods of 7 weeks. Furthermore, the previous studies were conducted in 25-week-old Hy-Line Brown hens [27], 28-week-old Lohmann Brown Classic hens [28], and 43- and 51-week-old Single Comb White Leghorn hens [26], whereas the present study monitored laying performance from 23 to 40 weeks of age, thereby covering a large part of the onset and early peak laying period, in LSL classic hens.

Egg weight and daily egg mass were generally similar among treatment groups throughout the study. Although daily egg mass was numerically greater in the HI-fed groups, the experimental design does not allow firm conclusions regarding whether this pattern reflects a true dietary effect or normal pen-to-pen variation. Nevertheless, the absence of any indication of reduced performance agrees with previous research reporting neutral or positive effects of HI-derived feed ingredients in laying hens, including the results of a meta-analysis [26,27,28,29,30].

The slightly greater feed intake observed in the HI groups should likewise be interpreted cautiously. Whether this pattern reflects a dietary response or normal variation among pens cannot be established from the present data.

The generally high digestibility of HI fat may partly explain the absence of adverse effects on production performance. Previous studies have shown that digestibility of HI fat is comparable to that of soybean oil in poultry and only slightly lower in rodents [17,40]. Collectively, evidence from the literature and the observations obtained in the present study suggest that complete replacement of soybean oil with HI fat is feasible under the investigated conditions. However, because production performance in our study was assessed in a single pen per treatment, these observations should be regarded as exploratory and require confirmation in replicated studies. 

### 4.2. Egg Quality Parameters

Under the conditions of the present study, no obvious differences were observed in major egg quality traits among the dietary treatments. Although some differences were detected at individual sampling points, these effects were small and inconsistent over time. For example, at 25 weeks of age, albumen height and Haugh units were slightly higher in the HI-50 group than in the HI-100 group, while the control group showed intermediate values. Since these differences were not observed at later sampling points, they are more likely to reflect transient variation than a biologically meaningful dietary effect. This interpretation is consistent with previous studies reporting no detrimental effects of HI fat on albumen quality or shell strength in laying hens [34,35,36]. Similarly, dietary supplementation with peppermint oil in laying quail altered lipid-related parameters without causing major changes in egg production or most egg quality characteristics [41], further supporting the notion that modifications of dietary lipid sources do not necessarily translate into pronounced changes in egg quality.

Yolk color was modestly increased in the HI-100 group at 30 and 35 weeks of age. Comparable effects have been reported previously when soybean oil was replaced by HI fat [26,27]. This response has been attributed to pigments naturally present in HI larvae, including carotenoids and melanin-related compounds that may be transferred to the yolk. However, the magnitude of the effect was small and likely of limited practical relevance, particularly because the diets used in the present study did not contain supplemental pigmentation sources. Under commercial conditions, yolk color is typically adjusted through supplementation with carotenoid-containing feed additives, which are likely to have a much greater impact on yolk pigmentation than the dietary fat source.

Similar conclusions have been drawn from studies using HI meal as a protein source, which also contains residual amounts of insect fat and generally does not impair egg quality characteristics [26,42]. Taken together, the available evidence does not indicate major adverse effects of HI-derived lipid sources on egg quality traits. Nevertheless, given the still limited number of studies and the lack of replicated experimental units in the present trial, further research is required to confirm these findings and to establish the robustness of the observed responses under commercial production conditions.

### 4.3. Fatty Acid Composition and Concentrations of Lipids (Triglycerides, Cholesterol) in Egg Yolks

The fatty acid profile of the HI fat used in the present study, characterized by a high proportion of lauric acid (C12:0), is consistent with previous reports on black soldier fly larval fat. However, it is well established that the lipid composition of HI larvae can vary considerably depending on the rearing substrate [7,13,43,44,45]. The predominance of medium-chain saturated fatty acids clearly distinguishes HI fat from conventional vegetable oils and largely explains the changes observed in yolk lipid composition. The close relationship between dietary fatty acid supply and yolk fatty acid composition has been demonstrated repeatedly in laying hens [46,47,48].

Although HI fat provided substantial amounts of lauric acid, its concentration in yolk lipids remained comparatively low. Similar observations have been reported in previous studies using HI fat in layer diets [27,49]. Heuel et al. [49] estimated that only about 3% of dietary lauric acid is deposited in the yolk. This limited transfer is most likely related to the metabolic fate of medium-chain fatty acids, which are preferentially oxidized for energy production rather than incorporated into body tissues or egg lipids [50,51].

As expected, replacing soybean oil with HI fat reduced the proportions of linoleic acid, α-linolenic acid, docosahexaenoic acid (DHA), and total PUFA in yolk lipids, while increasing the proportion of saturated fatty acids. These changes directly reflect the substantially lower PUFA content and different fatty acid spectrum of HI fat compared with soybean oil. Comparable shifts in yolk fatty acid composition have consistently been observed when HI fat or HI meal is included in layer diets [27,49,52]. This pattern is consistent with findings from a recent meta-analysis in broilers, which demonstrated that inclusion of black soldier fly larva meal can modify lipid composition and related physiological characteristics of animal products [16]. Together, these observations indicate that BSF-derived feed ingredients exert predictable effects on lipid deposition across different poultry production systems.

The observed increases in yolk saturated fatty acids and decreases in yolk polyunsaturated fatty acids can be largely explained by hepatic lipid metabolism in laying hens. In birds, the liver is the principal site of de novo fatty acid synthesis and the production of very-low-density lipoproteins (VLDL), which serve as the major lipid carriers for yolk deposition [53]. Because the fatty acid composition of VLDL is influenced by both dietary fatty acid supply and hepatic fatty acid metabolism, alterations in dietary fat sources are readily reflected in yolk lipids [31,32,46,47,48]. The increase in yolk saturated fatty acids observed in the present study can largely be explained by the high concentrations of lauric, myristic, and palmitic acid in HI fat. In addition, dietary fatty acids may affect the activity of hepatic desaturase enzymes, particularly stearoyl-CoA desaturase, which converts saturated fatty acids into monounsaturated fatty acids [31,32,54]. The increased proportion of palmitoleic acid (C16:1) in yolk lipids of the HI-fed hens may indicate enhanced desaturation of palmitic acid within the liver. Since yolk lipids are deposited through a highly regulated process involving hepatic lipoprotein synthesis, transport, and follicular uptake, the observed changes in yolk fatty acid composition likely reflect both direct dietary effects and metabolic adaptation of the liver to the altered fatty acid supply.

From a human nutritional perspective, the observed changes in yolk fatty acid composition deserve consideration. Replacement of soybean oil with HI fat increased the proportion of lauric acid which differs metabolically from longer-chain fatty acids. It is rapidly oxidized in the liver and is less readily incorporated into body lipid stores [50,51,55]. Replacement of soybean oil with HI fat also resulted in a marked increase in the overall proportion of saturated fatty acids and a concomitant reduction in both n-3 and n-6 PUFA. This shift may be regarded less favorably from a nutritional perspective because replacement of SFA by PUFA has been shown to improve plasma lipid profiles in humans, particularly through reductions in total and LDL cholesterol concentrations [56,57]. Moreover, n-3 PUFA contribute to cardiovascular health and the regulation of inflammatory processes [58,59,60]. Therefore, the observed increase in SFA together with the reduction in PUFA may reduce the nutritional value of eggs from a cardiovascular perspective, although the magnitude of such effects in the context of overall dietary intake remains uncertain.

### 4.4. Number and Size of Ovarian Follicles

In the present study, no obvious differences in follicle number or follicle size distribution were observed among treatments, suggesting that follicular development was not markedly altered by HI fat under the conditions of the present study. Yolk precursors, primarily VLDL and vitellogenin, are synthesized in the liver and subsequently deposited in developing follicles under estrogenic control [32]. Because hepatic lipid synthesis and follicular growth depend on an adequate supply of metabolizable energy and essential nutrients, including amino acids and essential fatty acids [32], the absence of treatment effects suggests that replacing soybean oil with HI fat did not compromise the nutritional support required for normal ovarian function. Nevertheless, direct measurements of energy metabolism and nutrient status were not performed, and this interpretation therefore remains indirect. The similar follicular populations observed among treatments are consistent with the descriptive production data, which did not reveal obvious differences in laying performance among groups. As the number of preovulatory follicles is closely related to ovulation rate and egg production [61,62], the similar follicular characteristics observed among treatments suggest that no major alterations in ovarian development were evident based on the measured endpoints. Although soybean oil and HI fat differ substantially in their fatty acid profiles, these differences did not translate into measurable changes in reproductive traits. This observation agrees with previous evidence indicating that, while dietary fat sources can markedly alter yolk fatty acid composition, reproductive performance in laying hens is generally maintained provided that essential fatty acid requirements are met [46,47,48].

The apparent resilience of follicular development to dietary fat source may reflect the fact that ovarian function is regulated predominantly by endocrine and intra-ovarian signaling pathways. In particular, granulosa cell proliferation, differentiation, and steroidogenesis are primarily controlled by hormonal signals rather than by variations in individual dietary fatty acids [63]. Taken together, the present findings indicate that replacement of soybean oil with HI fat was not associated with obvious changes in ovarian follicle characteristics. However, because reproductive function was evaluated only by follicle number and size and because hens were housed in a single pen per treatment, broader conclusions regarding reproductive function should be drawn with caution. In addition to the lack of replicated pens, the generalizability of the present findings is limited by the fact that the study was conducted using a single commercial layer strain (LSL Classic), during one production phase, and under a single set of housing and management conditions. Consequently, extrapolation of the results to other layer genotypes, age periods, and commercial production systems should be made with caution. Further studies conducted under different management conditions and with replicated experimental units are required to establish the broader applicability of the present findings.

## 5. Conclusions

The present study suggests that soybean oil can be partially or completely replaced by HI fat in diets for laying hens without apparent adverse effects on feed intake, laying performance, egg quality, or ovarian follicle development. The observed modifications in yolk fatty acid composition closely reflected the fatty acid profile of the dietary lipid source and were consistent with findings from previous studies using black soldier fly-derived feed ingredients.

A major limitation of the study is the absence of replicated pens, which restricts statistical inference and limits the generalizability of the findings. Consequently, the results should be regarded as exploratory and hypothesis-generating rather than confirmatory. Future studies employing replicated experimental units, additional layer genotypes, and different production conditions are required to confirm the present observations and establish their applicability under commercial production settings.

## Figures and Tables

**Table 1:** Composition and nutrient concentrations of the experimental diets.

	Phase 1			Phase 2		
	Control	HI-50	HI-100	Control	HI-50	HI-100
**Component (g/kg)**						
Wheat	163	163	163	162	146.2	148
Corn	285.4	292.8	297.5	274.5	291.1	290.8
Wheat bran	100	95	85.6	120	117.5	116
Soybean meal	221	213.5	221	210	210	210
Corn gluten meal	44	45.5	39	40	38	34.8
HI fat	0	36.2	72.5	0	36.2	72.5
Soybean oil	65	32.5	0	65	32.5	0
Mineral premix ^1^	20	20	20	20	20	20
Calcium carbonate	85	85	84.5	97	97	96.5
Monocalcium phosphate	6.5	6	6.1	3	2.8	2.5
Sodium chloride	1.8	1.9	2	1.1	1.1	1.1
L-Lysine	1.2	1.5	1.3	1.5	1.5	1.5
DL-Methionine	1.6	1.6	1.6	1.2	1.3	1.3
L-Valine	2.5	2.5	2.8	2.7	2.7	2.9
L-Threonine	1.3	1.3	1.4	0.5	0.6	0.6
L-Isoleucine	1.7	1.7	1.7	1.5	1.5	1.5
**Analyzed nutrient and calculated energy content**						
AME_N_ (MJ/kg)	11.4	11.4	11.4	11.1	11.1	11.1
Crude protein (%)	18.1	18.1	18.8	18.3	18.1	18.9
Ether extract (%)	8.85	9.12	8.76	8.77	8.39	8.58
Crude fiber (%)	3.15	3.42	3.96	3.69	3.24	3.95
Crude ash (%)	12.2	12.2	11.6	13.3	13.0	12.5
Starch (%)	30.8	31.0	30.0	30.3	31.1	31.2
Sugar (%)	3.15	3.24	3.33	3.25	3.15	3.24
Calcium (%)	3.94	4.01	4.15	4.14	4.21	4.34
Phosphorus (%)	0.84	0.82	0.81	0.78	0.77	0.75
**Amino acids (g/kg diet)**						
Methionine + cystein	8.03	7.91	8.24	7.17	7.26	7.19
Lysine	9.21	9.26	9.55	8.28	8.44	8.40
Threonine	7.71	7.90	8.00	5.71	5.92	5.85
Tryptophan	1.65	1.54	1.58	1.51	1.49	1.49
**Fatty acids (g/100 g of total fatty acids) ^2^**						
C12:0	0.09	11.5	29.9	0.02	10.1	30.8
C14:0	0.17	3.45	7.58	0.12	3.24	6.86
C14:1	<0.01	0.06	0.14	<0.01	0.06	0.14
C16:0	11.1	12.9	14.8	11.0	13.2	13.7
C16:1 n-7	0.06	0.91	1.91	0.07	0.90	1.71
C18:0	4.15	3.31	2.20	4.43	3.31	2.02
C18:1 n-7 + n-9	22.5	18.8	13.2	24.1	19.2	13.2
C18:2 n-6	54.3	43.2	27.0	53.5	44.5	28.3
C18:3 n-3	6.22	4.36	1.90	5.75	4.30	1.89
C20:0	0.43	0.24	0.19	0.47	0.33	0.18
C20:1 n-9	0.27	0.24	0.20	0.29	0.26	0.19

^1^ The mineral and vitamin mixture supplied the following per kg diet: Fe (Iron-II-sulphate monohydrate), 50 mg; Cu (Copper-II-sulphate pentahydrate), 12 mg; Mn (Manganese-II-oxide), 80 mg; Zn (Zinc oxide), 80 mg; Se (Sodium selenite), 0.32 mg; I (calcium iodate), 1.60 mg; vitamin A (Retinyl acetate), 10,000 IU; vitamin D3 (Cholecalciferol), 2000 IU; vitamin E (all-rac-α-Tocopheryl acetate), 30 mg; vitamin K3 (Menadione sodium bisulfite), 2.6 mg; vitamin B1, 2.0 mg; vitamin B2, 6.0 mg; vitamin B6, 3.2 mg; folic acid, 0.65 mg; nicotinic acid, 34.2 mg; pantothenic acid, 9.7 mg; biotin, 0.07 mg; vitamin B12, 0.026 mg; choline chloride, 400 mg; vitamin C (L-ascorbic acid), 150 mg. ^2^ Only fatty acids > 0.1% of total fatty acids are shown. HI fat, Hermetia illucens (HI) larvae fat; AME_N,_ nitrogen-corrected apparent metabolizable energy.

**Table 2:** Body weight at 18 (housing) and 40 weeks of age and body weight gain of laying hens fed either a conventional layer diet (Control) or diets in which 50% (HI-50) or 100% (HI-100) of the fat fraction was replaced with *Hermetia illucens* (HI) larvae fat.

Parameter	Control	HI-50	HI-100	*p*-Value
Body weight at week 18 (g)	1176 ± 63	1184 ± 57	1170 ± 49	0.722
Body weight at week 40 (g)	1756 ± 102	1778 ± 80	1790 ± 102	0.469
Body weight gain (g)	580 ± 108	605 ± 82	620 ± 94	0.405

Data are presented as means ± standard deviations; *n* = 20. All hens within a treatment were housed in a single compartment.

**Table 3:** Egg production, egg weight, daily egg mass production, feed intake, and feed conversion ratio of laying hens fed either a conventional layer diet (Control) or diets in which 50% (HI-50) or 100% (HI-100) of the fat fraction was replaced with *Hermetia illucens* (HI) larvae fat from 23 to 40 weeks of age.

Parameter	Control	HI-50	HI-100
Egg production (%)	94.8	97.2	98.1
Egg weight (g)	59.9	59.6	59.3
Egg mass (g/hen/day)	57.0	58.0	58.3
Feed intake (g/hen/day)	119	126	127
Feed conversion ratio	2.11	2.18	2.16

Data are presented as mean values derived from cumulative performance data recorded throughout the experimental period (23–40 weeks of age); *n* = 20. All hens within a treatment were housed in a single compartment.

**Table 4:** Albumen height, Haugh units, shell breaking strength, and yolk color of eggs collected at days 25, 30, 35 and 40 weeks of age from laying hens fed either a diet with soybean oil as source of fat (Control) or diets in which 50% (HI-50) or 100% (HI-100) of soybean oil was replaced with *Hermetia illucens* (HI) larvae fat.

Parameter	Control	HI-50	HI-100	*p*-Value
**Week 25 of age**				
Albumen height (mm)	7.14 ± 1.05 ^ab^	7.52 ± 0.82 ^a^	6.79 ± 0.61 ^b^	0.015
Haugh units	85.0 ± 6.8 ^ab^	88.3 ± 5.2 ^a^	83.9 ± 4.6 ^b^	0.038
Shell breaking strength (N)	53.2 ± 8.1	56.2 ± 6.1	55.8 ± 7.8	0.410
Yolk color score	5.58 ± 0.96	5.39 ± 0.70	5.53 ± 0.51	0.733
**Week 30 of age**				
Albumen height (mm)	6.38 ± 0.75	6.38 ± 0.75	6.28 ± 0.54	0.862
Haugh units	79.8 ± 4.6	79.3 ± 6.0	79.6 ± 3.9	0.822
Shell breaking strength (N)	51.7 ± 10.6	54.6 ± 10.2	57.3 ± 7.7	0.109
Yolk color score	5.16 ± 0.83 ^b^	5.37 ± 0.68 ^ab^	5.90 ± 0.91 ^a^	0.021
**Week 35 of age**				
Albumen height (mm)	5.69 ± 0.76	5.65 ± 0.91	5.39 ± 0.69	0.429
Haugh units	73.3 ± 6.2	73.0 ± 7.2	71.7 ± 6.0	0.715
Shell breaking strength (N)	54.9 ± 10.3	56.7 ± 8.6	55.1 ± 8.1	0.816
Yolk color score	4.10 ± 0.64 ^b^	4.32 ± 0.75 ^ab^	4.65 ± 0.59 ^a^	0.047
**Week 40 of age**				
Albumen height (mm)	6.73 ± 0.86	6.81 ± 0.75	6.87 ± 0.62	0.846
Haugh units	80.2 ± 5.0	81.4 ± 5.1	81.8 ± 4.7	0.556
Shell breaking strength (N)	53.2 ± 8.9	52.6 ± 9.9	49.2 ± 9.9	0.380
Yolk color score	5.21 ± 0.54	5.32 ± 0.75	5.11 ± 0.66	0.544

Data are presented as means ± standard deviations. Means within a row without common superscript letters (a, b) differ significantly (*p* < 0.05). *n* = number of eggs per treatment, depended on daily laying performance, *n* = 18–20. All hens within a treatment were housed in a single compartment.

**Table 5:** Fatty acid composition of eggs yolk total lipids from laying hens fed either a diet with soybean oil as source of fat (Control) or diets in which 50% (HI-50) or 100% (HI-100) of soybean oil was replaced with *Hermetia illucens* (HI) larvae fat collected at weeks 30 and 40 of age.

Fatty Acid	Control	HI-50	HI-100	*p*-Value
**g/100 g total fatty acids** **Week 30**				
C12:0	0.0863 ± 0.0423 ^c^	0.457 ± 0.050 ^b^	1.06 ± 0.22 ^a^	<0.001
C14:0	0.319 ± 0.094 ^c^	2.30 ± 0.28 ^b^	4.95 ± 0.70 ^a^	<0.001
C16:0	23.7 ± 0.8 ^c^	26.2 ± 0.5 ^b^	27.9 ± 1.0 ^a^	<0.001
C18:0	9.20 ± 1.05 ^a^	8.39 ± 0.66 ^ab^	7.61 ± 0.88 ^b^	0.003
Σ SFA	34.2 ± 1.1 ^c^	38.9 ± 0.8 ^b^	42.9 ± 1.2 ^a^	<0.001
C16:1 n7 + n9	1.98 ± 0.36 ^c^	2.86 ± 0.41 ^b^	4.70 ± 0.61 ^a^	<0.001
C18:1 n7 + n9	32.5 ± 2.2	31.0 ± 0.7	31.0 ± 1.9	0.094
Σ MUFA	35.0 ± 2.4 ^b^	34.7 ± 0.9 ^b^	37.2 ± 1.5 ^a^	0.005
C18:2n-6	25.6 ± 2.7 ^a^	22.4 ± 1.4 ^b^	16.8 ± 1.0 ^c^	<0.001
C20:4n-6	1.24 ± 0.09 ^a^	0.691 ± 0.187 ^b^	0.512 ± 0.097 ^c^	<0.001
C18:3n-3	1.31 ± 0.20 ^a^	0.984 ± 0.064 ^b^	0.631 ± 0.165 ^c^	<0.001
C22:6n-3	0.408 ± 0.042 ^a^	0.162 ± 0.067 ^b^	0.105 ± 0.035 ^c^	<0.001
Σ PUFA	30.8 ± 2.9 ^a^	26.4 ± 1.2 ^b^	19.9 ± 1.5 ^c^	<0.001
Σ n-6	28.3 ± 2.8 ^a^	24.2 ± 1.2 ^b^	18.4 ± 1.2 ^c^	<0.001
Σ n-3	2.56 ± 0.22 ^a^	2.16 ± 0.52 ^a^	1.46 ± 0.49 ^b^	<0.001
n-6/n-3	11.1 ± 1.1	11.8 ± 2.8	13.6 ± 3.3	0.136
**Week 40**				
C12:0	0.0576 ± 0.0164 ^c^	0.400 ± 0.053 ^b^	0.885 ± 0.230 ^a^	<0.001
C14:0	0.333 ± 0.040 ^c^	2.06 ± 0.24 ^b^	4.18 ± 0.55 ^a^	<0.001
C16:0	24.8 ± 0.8 ^c^	26.0 ± 0.9 ^b^	27.7 ± 1.2 ^a^	<0.001
C18:0	8.84 ± 0.80 ^a^	8.90 ± 0.68 ^a^	7.75 ± 0.59 ^b^	0.001
Σ SFA	35.9 ± 1.3 ^c^	39.0 ± 0.9 ^b^	42.1 ± 1.1 ^a^	<0.001
C16:1 n7 + n9	2.22 ± 0.27 ^c^	2.86 ± 0.22 ^b^	4.37 ± 0.75 ^a^	<0.001
C18:1 n7 + n9	33.5 ± 1.4	33.0 ± 1.2	32.5 ± 1.3	0.268
Σ MUFA	36.3 ± 1.3 ^b^	36.8 ± 1.1 ^b^	38.4 ± 1.3 ^a^	0.003
C18:2n-6	23.8 ± 1.8 ^a^	20.1 ± 1.2 ^b^	15.9 ± 1.6 ^c^	<0.001
C20:4n-6	0.466 ± 0.076	0.496 ± 0.063	0.426 ± 0.053	0.071
C18:3n-3	0.992 ± 0.152 ^a^	0.813 ± 0.133 ^b^	0.654 ± 0.138 ^b^	<0.001
C22:6n-3	0.113 ± 0.086 ^ab^	0.104 ± 0.019 ^a^	0.0770 ± 0.0127 ^b^	0.008
Σ PUFA	27.8 ± 2.2 ^a^	24.1 ± 1.6 ^b^	19.6 ± 1.9 ^c^	<0.001
Σ n-6	25.5 ± 1.9 ^a^	21.8 ± 1.3 ^b^	17.6 ± 1.7 ^c^	<0.001
Σ n-3	2.30 ± 0.86	2.27 ± 0.57	1.98 ± 0.50	0.506
n-6/n-3	12.2 ± 3.6	10.2 ± 2.5	9.39 ± 2.62	0.113

Data are presented as means ± standard deviations; *n* = 10 eggs per treatment and sampling point. All hens within a treatment were housed in a single compartment. Means within a row without common superscript letters (a–c) differ significantly (*p* < 0.05). MUFA = monounsaturated fatty acids, PUFA = polyunsaturated fatty acids, SFA = saturated fatty acids.

**Table 6:** Egg yolk triglyceride and cholesterol content of eggs from laying hens fed a diet with soybean oil as source of fat (Control) or diets in which 50% (HI-50) or 100% (HI-100) of soybean oil was replaced with *Hermetia illucens* (HI) larvae fat collected at weeks 30 and 40 of age.

Parameter	Control	HI-50	HI-100	*p*-Value
**mg/g egg yolk** **Week 30**				
Triglycerides	164 ± 7 ^a^	157 ± 11 ^ab^	151 ± 9 ^b^	0.019
Cholesterol	14.9 ± 0.3 ^a^	13.4 ± 0.5 ^b^	14.5 ± 1.0 ^a^	<0.001
**Week 40**				
Triglycerides	157 ± 17	152 ± 10	159 ± 16	0.627
Cholesterol	14.1 ± 1.0	13.8 ± 0.7	14.2 ± 1.0	0.630

Data are presented as means ± standard deviations; *n* = 10 eggs per treatment and sampling point. All hens within a treatment were housed in a single compartment. Means within a row without common superscript letters (a, b) differ significantly (*p* < 0.05).

## Data Availability

The original data presented in the study are openly available in a repository [https://doi.org/10.5281/zenodo.21903586].
